# Combining Targeted Metabolomic Data with a Model of Glucose Metabolism: Toward Progress in Chondrocyte Mechanotransduction

**DOI:** 10.1371/journal.pone.0168326

**Published:** 2017-01-05

**Authors:** Daniel Salinas, Cody A. Minor, Ross P. Carlson, Carley N. McCutchen, Brendan M. Mumey, Ronald K. June

**Affiliations:** 1 Computer Science, Montana State University, Bozeman, MT United States of America; 2 Mathematics, Montana State University, Bozeman, MT United States of America; 3 Chemical & Biological Engineering, Montana State University, Bozeman, MT United States of America; 4 Mechanical & Industrial Engineering, Montana State University, Bozeman, MT United States of America; 5 Department of Cell Biology & Neurosciences, Montana State University, Bozeman, MT United States of America; 6 Department of Orthopaedics and Sports Medicine, University of Washington, Seattle, WA United States of America; Mayo Clinic Rochester, UNITED STATES

## Abstract

Osteoarthritis is a debilitating disease likely involving altered metabolism of the chondrocytes in articular cartilage. Chondrocytes can respond metabolically to mechanical loads via cellular mechanotransduction, and metabolic changes are significant because they produce the precursors to the tissue matrix necessary for cartilage health. However, a comprehensive understanding of how energy metabolism changes with loading remains elusive. To improve our understanding of chondrocyte mechanotransduction, we developed a computational model to calculate the rate of reactions (*i*.*e*. flux) across multiple components of central energy metabolism based on experimental data. We calculated average reaction flux profiles of central metabolism for SW1353 human chondrocytes subjected to dynamic compression for 30 minutes. The profiles were obtained solving a bounded variable linear least squares problem, representing the stoichiometry of human central energy metabolism. Compression synchronized chondrocyte energy metabolism. These data are consistent with dynamic compression inducing early time changes in central energy metabolism geared towards more active protein synthesis. Furthermore, this analysis demonstrates the utility of combining targeted metabolomic data with a computational model to enable rapid analysis of cellular energy utilization.

## Introduction

Osteoarthritis (OA) is the most common joint disorder worldwide and involves the metabolic dysfunction of chondrocytes found in articular cartilage [1–5]. The National Health and Nutrition Examination Survey I found that 12.1% of the US population aged 25–74 years had OA in some joint [6]. While joint trauma increases the risk of OA, moderate exercise and associated mechanical loading are linked to improved joint health [7, 8]. For these reasons, understanding chondrocyte responses to mechanical stimulation can yield insight into the initiation, progression, and treatment of osteoarthritis.

Cartilage, a viscoelastic tissue, dissipates energy internally upon cyclical loading. Activities that deform cartilage, *e*.*g*. daily activity [9], have the potential to alter the energy metabolism of chondrocytes via mechanotransduction. We have previously observed changes in concentrations of central energy metabolites as a result of dynamic compression [10]. Other studies demonstrate that chondrocytes sense compression and can distinguish between static compression, dynamic compression, and even shear. Long-term chondrocyte mechontransduction responses (1–24 h) involve the expression of mRNA for extracellular matrix (ECM) and pericellular matrix (PCM) proteins [11, 12].

**Fig 1:**
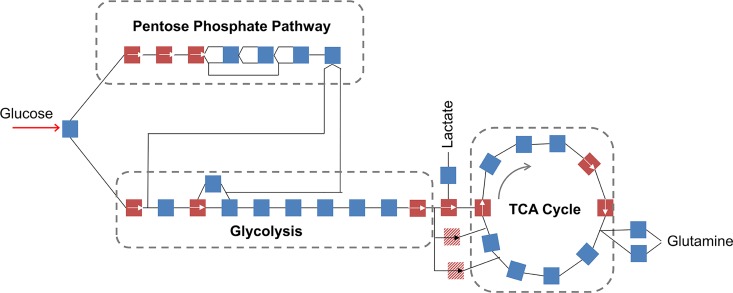
Mammalian central energy metabolism. Glucose metabolism, including the pentose phosphate pathway, glycolysis, and the TCA cycle, was modeled as a network using a stoichiometric matrix. Each colored square represents a biochemical reaction, and each line represents a metabolite. Reversible reactions shown in blue, and irreversible reactions shown in red. Arrows indicate the direction of irreversible reactions. Anaplerotic reactions that replenish TCA reactants are represented by hashed squares. The curved arrow indicates the direction of normal flux in the TCA cycle. Selected metabolites (*i*.*e*.glucose, lactate, and glutamine) shown explicitly.

Cartilage is known to be viscoelastic, indicating that cycles of load (*e*.*g*. walking) result in energy dissipation to the tissue [13]. We hypothesized physiological compression of chondrocytes would cause central energy metabolism (Fig 1) to increase production of amino acid precursors for ECM and PCM proteins as an early-time (<30 min) response. Because early-time behavior (*e*.*g*. metabolic changes) sets the trajectory for longer-term responses (*e*.*g*. matrix synthesis), we measured changes in central energy metabolites resulting of dynamic compression over 0–30 minutes [14].

Here, we apply metabolic flux analysis to quantify fluxes through the individual reactions of central metabolism. We first construct a stoichiometric matrix that quantitatively accounts for the catabolism of glucose within glycolysis, the pentose phosphate pathway, and the TCA cycle. This matrix provides a tool for calculating the individual reaction rates (*i*.*e*. fluxes) in central metabolism based on targeted metabolomic data. This model consists of glycolysis (G), pyruvate processing into acetyl-CoA (PYP), the tricarboxylic acid cycle (TCA), the pentose phosphate pathway (PPP), the electron transport chain (ETC), the anaplerotic (AP) reactions, and lactate (LDH) and (GDH) glutamine dehydrogenases (Fig 1). Stoichiometric modeling has been used to examine molecular-level phenomena, including cellular phenotypes. Stoichiometric models do not require extensive knowledge of enzyme kinetic parameters, data that is often very difficult to acquire *in vivo*. Previous applications of these models examined physiologies of model systems ranging from viruses, to individual microbial cells, to microbial communities, to human tissues [15–19].

The model developed in this study allowed us to estimate the allocation of resources such as glucose and its derivatives. We calculated average flux profiles in response to 0–30 minutes of physiological loading. Results show a sustained increase in respiration in compressed chondrocytes. Upon further compression to 30 minutes, fluxes show increased glycolysis in compressed cells accompanied by depletion of metabolites used in protein synthesis. These data highlight the importance of energy metabolism in the chondrocyte response to mechanical loading and suggest protein synthesis as a consequence. To our knowledge, this is the first systems analysis of chondrocyte energy metabolism in response to compression. By fitting the stoichiometric model to experimental data [20], we obtain a holistic view of how glucose metabolism is altered during compression.

## Materials and Methods

### Chondrocyte compression

*In vivo* chondrocytes reside within a pericellular matrix composed of type VI collagen and perlecan [21] that serves as the primary source for delivering deformational stimuli. Loading chondrocytes *in vitro* required encapsulating them in a material that successfully emulates how cartilage transmits loads. A major challenge to *ex vivo* studies of chondrocyte mechanotransduction has been replicating the mechanical stiffness of the PCM (between 25–200 kPa [22, 23]). Accurately modeling deformations is important as they are known to affect chondrocyte biology [24]. For example, the PCM can release FGF2, a fibroblast growth factor, to initiate diverse signaling in response to external stimuli [25]. This study used previously published data involving SW1353 chondrocytes subjected to compression in agarose of physiological stiffness [10, 14]. SW1353 cells were selected because they are a commonly used in chondrocyte and osteoarthritis research [26–28].

**Fig 2:**
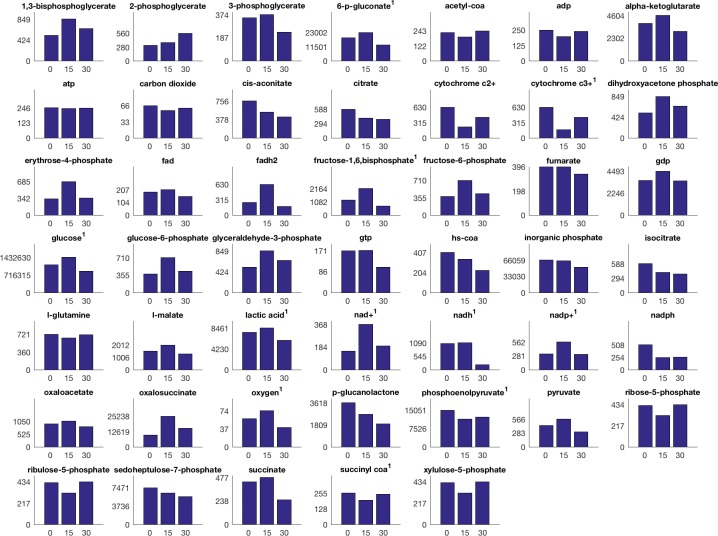
Median metabolite LC-MS intensities over time and experimental group. Colors indicate sample time, from left to right in the group 0,15, and 30 minutes. Superscript numbers indicate the probability of no difference between population means is less than or equal to p = 0.15, as calculated by ANOVA. P-values for all metabolites are found in the supplementary material (S2). All intensities scaled by 0.001 for ease of display.

Encapsulated chondrocytes were then divided into experimental groups for 0 (control), 15, and 30 minutes of compression. Chondrocytes of the experimental groups were exposed to compression simulating the human gait; namely, sinusoidal compression with 1.1 Hz frequency oscillating between 3.1–6.9% strain. The abundance of targeted metabolites in samples was measured using LC-MS and has been previously published [8]. Here, we updated these methods by performing the chromatography with a HILIC column that enabled detection of additional relevant metabolites such as lactate (Fig 2).

### Metabolic flux analysis

Abundance measurements can be used to estimate the accumulation rate Δ_i_ of a metabolite i by

$$\Delta_{i}=\frac{a_{1}-a_{0}}{t_{1}-t_{0}}$$ (1)

where a_1_,a_0_ are the average of the abundance samples taken at time t_1_,t_0_ respectively. If the sample size is small the median can be used to curtail the influence of outliers. This was the case for our study, where the sample size was n = 4 or 5.

Given a vector of accumulation rates for all metabolites in the network (Δ), the average reaction fluxes (*v*) from *t*_*0*_ to *t*_*1*_ can be calculated by solving the system

Sv=Δ (2)

for *v*, where *S* is the stoichiometric matrix for chondrocyte central metabolism.

A system with a unique solution will have a stoichiometric matrix of full rank. Most biological models are rank-deficient, with an infinite number of solutions if the problem is feasible [29].

To find a unique solution representing empirically calculated fluxes for a given experiment, we measure metabolite consumption and accumulation rates during the experiment to account for some of the unknown fluxes. This can make the system determined and therefore uniquely solvable. It is typical to assume all internal metabolites have a zero accumulation inside the cell (Δ = 0) [30].

We opted for Eq 1 as a best estimate because ANOVA, using duration of compression as a factor f, showed a likely change in concentration (p *≤* 0.15) in 10 of the 45 metabolites measured [S2]. This may be expected for chondrocytes undergoing a metabolic shift.

Solutions to Eq 2 may include negative fluxes for reactions thermodynamically feasible only in the forward direction. A bounded variable least squares problem (BVLS) approximates v while respecting thermodynamic constraints. The problem objective is minimizing the residual magnitude over *v*

$$\min_{v}{\left\|Sv-\Delta \right\|}_{2}$$ (3)

with constraints on *v* to enforce a positive flux for irreversible reactions. This is expressed as linear inequalities *v*_*i*_ ≥ 0 where v_i_ is the flux of an irreversible reaction *i*.

To account for measurement uncertainty, we weight each element of the residual according to the variance of the corresponding metabolite accumulation. The variance of the accumulation of a single metabolite *i* (*σ*_*i*_^*2*^) is computed from the variances of the two samples used to calculate it (Eq 1). We estimate the variance of the difference in means between two samples with unequal variances with an established method [31]

$$\sigma_{i}^{2}=\frac{s_{1}^{2}}{n_{1}}+\frac{s_{2}^{2}}{n_{2}}$$ (4)

where *s*_*k*_, *n*_*k*_ are the standard deviation and size of sample *k*. The weight of metabolite *i* (*w*_*i*_) is the reciprocal of the variance

$$w_{i}=\sigma_{i}^{-2}$$ (5)

To apply weight *w*_*i*_, row *S*_*i*_ and Δ_*i*_ are both multiplied by *w*_*i*_. The resulting vector and matrix are both used to formulate the BVLS. We opted to solve the BVLS with an interior point method since this converges within polynomial time.

### Stoichiometric matrix construction

The reactions of central metabolism were encoded in a stoichiometric matrix [S1 Table] that was then adapted to the targeted metabolite data. The stoichiometric matrix is defined from established biochemistry in mammalian cells based on the input of a single molecule of glucose [32]. Calculating the accumulation of some metabolites was technically infeasible, either because the analytes were not detectable using positive-mode LC-MS (*e*.*g*. cytochrome, H+), or because the concentration in the media was so high the random difference between samples would likely be much larger than any change in cell activity would induce (*e*.*g*. glucose).

To find the exact solutions, we modified the full network to preserve pathways while using only measurable metabolites. Unmeasurable internal metabolite fluxes were constrained to be zero to preserve pathways while allowing reactions that involve measurable metabolites to balance the observed changes in abundance. Unmeasurable external metabolites were not included as they cannot reasonably be constrained to a value [S3 Table]. Of these, we make an exception for the unmeasurable ETC metabolites. Reduced and oxidized forms of ubiquinone, as well as the protons involved in the proton gradient across the mitochondrial inner membrane were the only unmeasurable metabolites included. These metabolites are all contained within the mitochondrial membrane and associated proteins, and the cell will tolerate very little change in their relative concentrations between reduced and oxidized forms [32]. The protons are not bound to the membrane or associated enzymes. In a close to neutral environment such as these *in vitro* data, most of this proton gradient contributes to a potential difference (as opposed to a pH gradient) Because the cell tolerates very little change in this reductive potential, the accumulation of these metabolites was assumed to be 0. The residuals for these metabolites were, however, given the lowest weight. The resulting matrix [S3 Table] had dimensions 48 by 39 (rank 39).

Finally, we use a synthesis reaction to account for central energy metabolites consumed as substrates in the production of PCM proteins. The proteins investigated were type II collagen, type VI collagen, and aggrecan; albumin and lipid were used as negative controls, since neither was expected to be produced by chondrocytes in substantial quantities. We focused on 3-phosphoglycerate, pyruvate, α-ketoglutarate, and oxaloacetate as designated precursors for proteins, and acetyl-CoA for lipids. Synthesis reactions are represented by a column with negative coefficients for the rows of precursor metabolites and zeros in all other rows [S1 Code]. The coefficients represent the ratiometric amount of each precursor consumed by the reaction to synthesize a single unit of its product. They were calculated from the amino acid sequence for each protein. We solved for fluxes with each of the six different synthesis reactions to find which synthesis reaction fit the data best.

### Exemplary flux vectors

Flux vectors that maximize flux through the synthesis reaction were calculated. These exemplary profiles represent metabolism that is mathematically optimized toward synthesis of a particular product and served as a point of reference for the experimental flux vectors. They are encoded as the solution to a linear program

$$\min_{v}c^{T}v$$ (6)

$${s.t.\;S}_{I}v=0$$ (7)

Av≥0 (8)

v≤1 (9)

where *c* is a vector of all zeroes except for a coefficient of -1 at the synthesis reaction index. The first constraint was added to ensure the solution consists of feasible pathways. *S*_*I*_ is the submatrix of *S* that consists of rows corresponding to internal metabolites. Internal metabolites serve as pathway intermediates from input to output metabolites, and, as such, their net accumulation is zero when the network is at steady state. The accumulations for external metabolites (*e*.*g*. glucose) are unconstrained as they are substrates or products of the network as a whole and are therefore not balanced internally [S6 Table]. Irreversibility constraints are encoded in the matrix *A*, where *a*_*ij*_ = 1 only if *i* = *j* and *v*_*i*_ is irreversible; all other elements are 0. Since the program is otherwise unbounded the final constraint limits the maximum flux of any reaction in *v* to 1.

**Fig 3:**
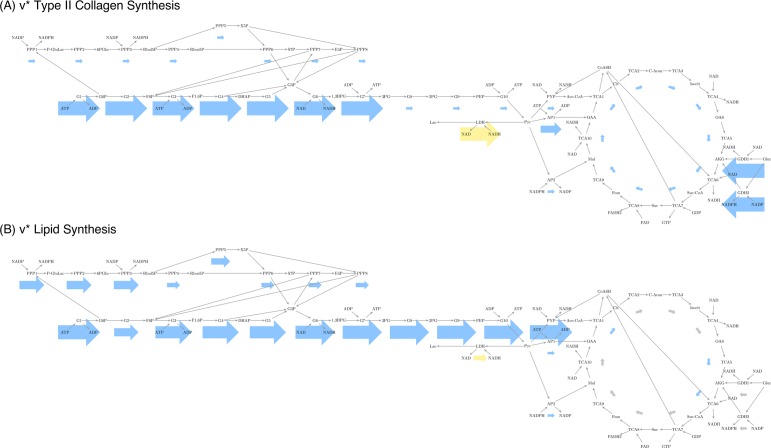
Hypothesis-based predicted flux profiles, v*. Flux profiles were predicted *in silico* using optimization to maximize production of (A) type II collagen and (B) lipid synthesis. Reactions are represented by labeled boxes connected to substrates by incoming dark gray arrows and to products by outgoing dark gray arrows. Reaction fluxes are represented by arrows colored to match either positive (blue) or negative (yellow) flux. Larger arrows represent larger flux with linear scaling. Negative flux indicates a reversible reaction running in the direction opposite to its description in the stoichiometric matrix. Electron transport chain and synthesis reactions not shown for figure simplicity.

Analysis of these exemplary profiles showed protein synthesis profiles were all similar. All proteins, including the generic negative control protein albumin, require the same precursors from central metabolism. However, the differing proportions were not different enough based on variations in the amino acid sequence to allow conclusive protein identification using exclusively central metabolism data [S1 Code, S6 Table]. Our hypothesis therefore only predicts compressed chondrocytes showing features common among profiles for protein synthesis, as the model is useful in identifying these in contrast to synthesis of other biological products, such as lipids (Fig 3).

Metabolic features of protein synthesis include high activity for glycolysis and the pentose phosphate pathways, and an uneven distribution of flux in the TCA cycle and glycolysis. The unevenness is caused by the loss of some intermediates as protein substrates. In glycolysis, 3-phosphoglycerate and pyruvate serve as precursors, while in the TCA cycle the precursors are oxaloacetate and and α-ketoglutarate. Both glycolysis and the TCA cycle show higher activity in the reactions before the precursors compared to the reactions after them. In contrast, lipid synthesis optimizes production of acetyl-CoA by maximizing flux through pyruvate processing; flux through glycolysis is also maximized to provide enough pyruvate for conversion. This is complemented by flux through the TCA cycle to generate the HSCoA consumed in acetyl-CoA synthesis. Since all metabolites used in these pathways are balanced except for acetyl-CoA, glycolysis and TCA flux distributions are more even.

## Results

**Fig 4:**
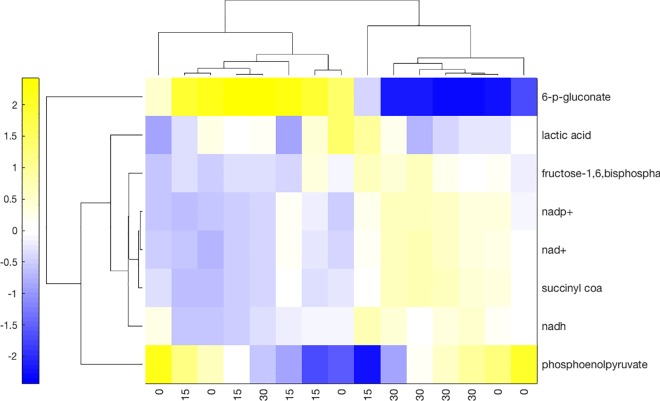
Metabolism Synchronization. Intensities of metabolites that changed significantly over time as indicated by ANOVA were used to cluster the samples. The distance between the samples is the correlation of their intensities. This distance was then used to cluster rows (intensity of a single metabolite over all samples) and columns (intensity of all metabolites belonging to a single sample). While the 15 and 0 minute samples have similarities, all but one of the 30 minute samples cluster together, indicating more similarity within 30 minute samples than to any other group. The intensities have been standarized by column.

Chondrocytes under compression seem to have distinct behavior in the first and second periods. After 30 minutes of compression, chondrocyte metabolomics profiles are also more similar to each other than to the other samples from another time, in contrast to the 15 and 0 minute samples. (Fig 4) This partially confirms our early-time hypothesis to the extent that compression has a measurable effect on chondrocytes in the short term. This was confirmed by the calculated flux vectors, which show a shift in metabolism occurs. Varying product synthesis reactions did not change the observed trends, even when using the lipid synthesis reaction [S5 Table]. This is especially true for the fluxes of the second 15 minute period, where the maximum distance between two vectors computed with differing synthesis reactions was less than 1% of the norm of the smaller vector. This may be due to a number of factors, such as the precursor metabolites receiving lower weight (except for 3PG, which tended to weight in the top half), or it may be indicative that no single profile dominated metabolism. For conciseness we show the visualization of fluxes calculated using the synthesis reaction for collagen type II (Fig 3) with the understanding that the figures for other synthesis reactions are similar.

**Fig 5:**
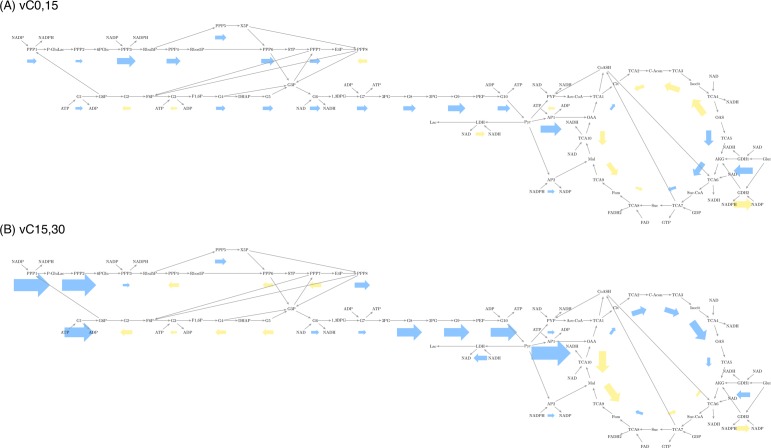
Experimentally-derived flux profiles for compressed chondrocytes display both similarities and differences to hypothetical predictions. Fluxes calculated for compressed cells for time intervals (A) 0 to 15 min. and (B) 15 to 30 min using the collagen synthesis reaction as described in the text. Similarities (in comparison to predictions in Fig 3A) include large relative fluxes in G1-3 and TCA1-3. Differences include small negative fluxes as discussed in the manuscript. Reactions are represented by labeled boxes connected to substrates by incoming dark gray arrows and to products by outgoing dark gray arrows. Reaction fluxes are represented by arrows colored to match either positive (blue) or negative (yellow) flux. Larger arrows represent larger flux with linear scaling. Negative flux indicates a reversible reaction running in the direction opposite to the direction specified in the stoichiometric matrix. Electron transport chain and synthesis reactions not shown for figure simplicity.

**Fig 6:**
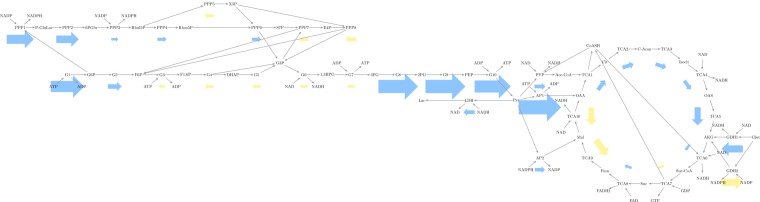
Overall flux trends highlight differences between compressed cells for time interval 0 to 30 min. Reactions are represented by labeled boxes connected to substrates by incoming dark gray arrows and to products by outgoing dark gray arrows. Fluxes are represented by arrows colored to match either positive (blue) or negative (yellow) flux. Larger arrows represent larger flux with linear scaling. Negative flux indicates a reversible reaction running in the direction opposite to the direction specified in the stoichiometric matrix. Electron transport chain and synthesis reactions not shown for figure simplicity.

During the first 15 minutes, compressed chondrocytes showed higher glycolysis and pentose phosphate flux than flux through the TCA cycle (Fig 5). During the second period, they showed an increase in TCA cycle flux, as well as increases in the pentose phosphate and glycolytic flux, though the increase was not uniform throughout the pathways. (Fig 6) There is a reversal in several glycolytic reactions, along with the pentose phosphate pathway reactions that interact with them. Glyceraldehyde-3-phosphate (G3P) links the two pathways. This metabolite increases during the first 15 minutes and subsequently decreases. This link is important since, as glyceraldehyde-3- phosphate dehydrogenase (GAPDH) can be upregulated by oxidative stress, which mechanical stimulation induces in chondrocytes, as elaborated in the Discussion section. This stress, as observed in our own computed fluxes, also affects the pentose-phosphate pathways. Finally, there is a persistent negative flux in the TCA cycle reactions between oxaloacetate and fumarate, though they follow the trend of increased magnitude in the second period.

**Fig 7:**
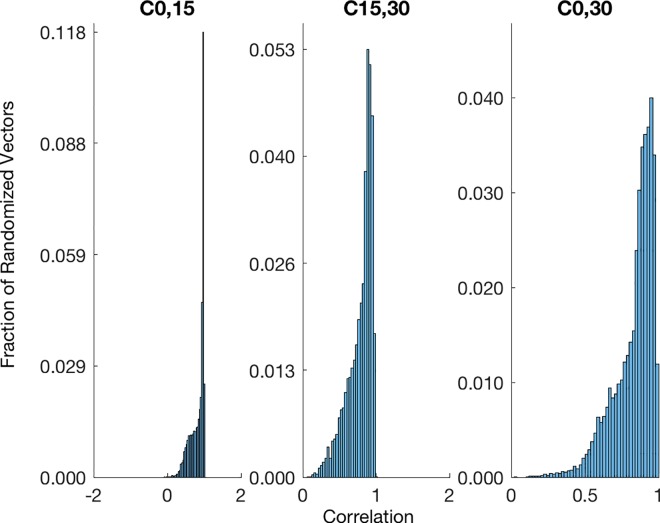
The majority of perturbed fluxes correlate with fluxes calculated from experimental data in a sensitivity analysis. Histogram of correlation values for 10000 randomized data sets compared with the empirical flux calculated for the unperturbed dataset. Each randomized data set yielded three randomized flux vectors which were then compared to the corresponding empirical flux vector calculated from the raw data.

To assess the variability of the fluxes, we conducted a sensitivity analysis. We created 100000 randomized data sets and compared the resulting fluxes to our original fluxes. The randomized sets were created by estimating the standard deviation of each sample for a given time and metabolite. This was used to compute the 95% confidence interval for the metabolite intensity. Normally distributed values within this 95% confidence interval were chosen as measurements. The fluxes were then solved as described above. To compare these randomized fluxes to our originals, we used the correlation as a similarity metric. Briefly, correlation is a scale-less metric with values from -1 (for opposite vectors) to (1 (equal vectors). A value of 0 can be interpreted as having no correlation. The histogram of correlation values shows that most of the randomized vectors had a high correlation (> 0.5). (Fig 7).

**Fig 8:**
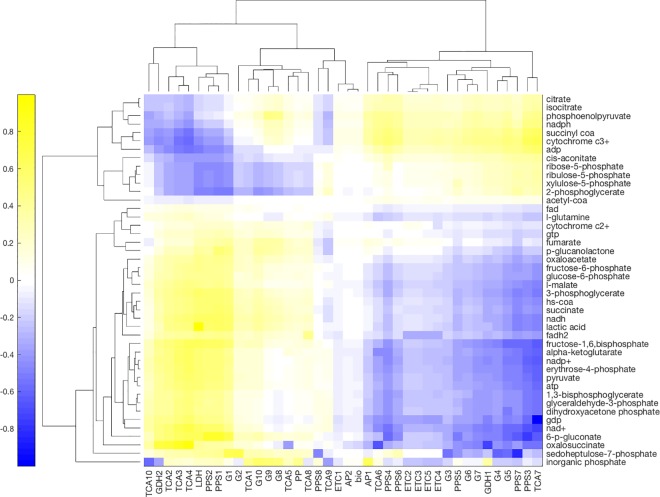
Pairwise correlation between calculated reaction flux and experimental metabolite accumulation. Positive correlation indicates that high flux is paired with high accumulation, and vice versa. Zero correlation indicates the flux cannot be predicted by the accumulation. Hierarchical clustering based on the Euclidean distance between fluxes and metabolite accumulation.

We also explored the relationship between metabolites and reactions. In this instance, we calculated the correlation between accumulation and resulting flux. We used the resulting correlation to cluster the reactions into groups that react similarly to changes in metabolite accumulations. We also clustered metabolites into groups that had similar effects on reaction fluxes. Both reactions and metabolites split into two groups. (Fig 8) The first group of reactions is composed mainly by the central pathways: glycolysis, TCA, and PPP reactions. The second contains most of the reactions that deal with external (no required to be balanced) metabolites: ETC, AP, the synthesis reaction, though it includes a few PPP and TCA reactions. This group correlates positively with a subset of the sugars in PPP. On the other hand, the main pathway metabolites correlate positively with the other, main pathway group. Interestingly, ATP correlates positively with the main metabolite group and ADP with the fringe group, possibly indicating two main reaction groups: ATP synthesis reactions and a profile, as observed in the data and described further in the Discussion, associated with reducing damage by ROS.

## Discussion

Calculating fluxes from experimental metabolomic data from compressed and uncompressed chondrocytes allows us to explore the short-term effects of an *in vitro* model of moderate exercise. We predicted features for flux profiles of chondrocytes undergoing protein synthesis using linear programming. While compressed chondrocytes showed some of these features, negative fluxes evinced metabolic shifts not accounted for by our hypotheses. The calculated fluxes captured behavior not predicted by the hypotheses generated in our linear programs, but upon further investigation proved these fluxes to be associated with metabolic behavior observed in SW1353 chondrosarcoma. This is, to our knowledge, is the first description in terms of reaction fluxes of chondrocyte response to exercise.

In this section we discuss possible causes for the negative fluxes as well as evidence of protein synthesis and other metabolic profiles by examining changes in metabolites (Fig 2), with significance values from two-way ANOVA [S2 Table], and applying known chondrocyte biology. During the first 15 minutes, compressed chondrocytes show metabolism geared toward glycolytic activity. Chondrocytes synthesize ATP primarily via glycolysis, and the high flux through glycolysis is consistent dovetails with the observed increase in the ratio of ATP to ADP. Thus, both ΔC0,15 and vC0,15 demonstrate substantial glycolytic ATP production, consistent with prior literature [33, 34]. This interpretation is supported by the experimental methods which included a tissue culture environment containing 4.5 g/L of glucose and by prior studies finding that cancer cells exhibit high glycolytic metabolism [35].

Metabolomic data also suggests compression initially induces higher TCA cycle activity as observed in vC0,15 and to a greater degree in v15,30 First, we observe that NADH, produced primarily in the TCA cycle, accumulates in ΔC0,15. The increase in the second period is corroborated by the depletion of citrate and cis-aconitate. Though higher intermediate volume is necessary for higher flux through a pathway, it is likely that these metabolites are not replenished as TCA cycle intermediates, e.g. AKG and OAA, are consumed as precursors to biomolecules, as previously discussed. In ΔC15,30 all four of the metabolites we identified as precursors (AKG,OAA,PYR,3PG) decrease in abundance. Alternatively, they chondrocytes may be engaged in higher respiration. There is some evidence for this occurring during the first 15 minutes of compression. Namely, a decrease in ADP levels coupled with a slight increase in NADH, as previously mentioned. Though high flux through the TCA cycle is unusual in chondrocytes, they are known to supplement ATP production through respiration under nutrient stress and mechanical loading [36–39]. Our analysis suggests compression may trigger the TCA cycle in an *in vitro* environment containing atmospheric oxygen levels, possibly for increased ATP synthesis, or for synthesis of pathway intermediates.

Coupled with an increase in TCA flux, both the pentose phosphate pathway and glycolysis experience an increase in the magnitude of their fluxes, though some of these fluxes reverse. As mentioned previously, the reactions that link to glyceraldehyde-3-phosphate (G3P) are the ones affected. Previous research has established a link between mammalian chondrocytes and the release of reactive oxygen species (ROS) by mitochondria as caused by mechanical stimulation [20]. A common mechanism is the upregulation of the pentose phosphate pathway to synthesize NADPH; this enables, among other things, the recycling of glutathione. [40] This is accompanied by a downregulation of GAPDH, or G6 in our model. As the consumption of G3P via G6 becomes impaired, the reversal of PPP observed is consistent with cancerous proliferation: namely, ribose-5-phosphate used as a precursor to nucleotides [41]

An explanation for negative TCA9-10 flux in vC0,15 and vC15,30 comes from examining the NAD+ and NADH. The reactants of TCA9-10, respectively l-malate and oxaloacetate, have high variances (moreover, NADH and NAD+ low variances) and are therefore weighted lower. Since forward flux through the TCA cycle consumes NAD+ and produces NADH, *in silico* reversal of TCA9-10 would balance the NADH and NAD+ at a lower cost in the residual than reversing other reactions. It should be noted, however, that since the Gibbs free energy is positive for forward activity of malate dehydrogenase, a reverse flux *in vivo* is not unrealistic [32]. *In vivo*, the ETC oxidizes NADH, producing NAD+, but its flux was low across all calculated profiles. This is consistent with the higher inhibition of ETC activity observed in cells with mutant isocitrate dehydrogenase (IDH), a common mutation in chondrosarcoma [42]. This strongly supports the utility of this stoichiometric model the flux balance analysis approach used in this study

Though pathway fluxes are generally consistent, exceptions show that our methodology may benefit by expanding our current model of chondrocyte central energy metabolism, larger sample sizes, and continuing our research on primary chondrocyte data instead of the SW1353 line currently used. Negative fluxes in TCA2-4 and TCA9-10 may have been caused by pathways active in chondrosarcoma not included in our chondrocyte model. As mentioned, the ETC presents low flux. However, NAD+ increases in ΔC0,15. These may cause negative fluxes in TCA2-4. This is consistent with chondrosarcoma abnormalities, specifically mutations in either IDH1 (cytosolic) or IDH2 (mitochondrial). IDH catalyzes the transformation of isocitrate to α-ketoglutarate and is responsive to NADP+ and isocitrate concentration [43]. NADP+ increases in abundance during the first fifteen minute interval. This may trigger IDH in that interval. However, the abundance of α-ketoglutarate does not increase significantly in the second interval as a result, and actually decreases. the uncompressed group. Mutant IDH consume α-ketoglutarate to produce (D)-2-hydroxyglutarate, and may be the reason α-ketoglutarate fails to accumulate significantly. SW1353 are known to have mutant IDH2 [44]. Mutant IDH2 regenerate NADP+ when generating (D)-2-hydroxyglutarate, however, implying some other process was keeping mitochondrial NADP+ from accumulating in the second time interval. In light of the peculiarities of SW1353 metabolism, future studies will utilize primary chondrocytes.

While metabolic flux analysis is a powerful tool to incorporate all abundance measurements simultaneously, the small sample size (n = 5) and computational techniques used for this study did not allow a conclusive answer to the question of whether production of specific proteins (e.g. type II collagen) could be detected following compression. This limitation results from biochemical fundamentals: single metabolites (*e*.*g*. 3-phosphoglycerate) are precursors to multiple amino acids, rendering non-unique mappings from specific metabolite fluxes to primary amino acid sequences. The current study allows us to describe metabolic trends; our future work will include an enlarged model and sample sizes to allow for more specific conclusions. Furthermore, SW1353 chondrocytes stem from chondrosarcoma tumor tissue. Cancer cells often have altered metabolism including substantially upregulated glycolysis [35]. Expansion of these methods to primary cells will provide further insight into chondrocyte glycolysis in osteoarthritic and normal chondrocytes.

Using metabolic flux analysis we have calculated reaction activities which correspond to observed disturbances in metabolite abundance. Using a bounded variable least squares problem allows us to consider all metabolic data simultaneously and objectively, while weighting data according to its reliability. SW1353 chondroctyes respond to compression by initially increasing both glycolysis and respiration and then subsequently lowering the flux through these pathways, consistent with protein synthesis and mechanotransduction. Future work utilizing additional data and an expanded model is planned.

## Supporting Information

S1 Table Stoichiometric matrix of central energy metabolism. The network consists of glycolysis, the TCA cyle, pentose phosphate pathway, electron transport chain, anaplerotic reactions, and transformation of pyruvate into acetyl-CoA. This file represents this network as a matrix with 38 columns representing the reactions and 52 rows representing the individual metabolites.
(XLSX)

S2 Table ANOVA examining the effects of compression time on metabolite abundance. Samples taken at 15 and 30 minutes. The table shows the P-values for the null hypothesis when analyzed by two-factor ANOVA.
(XLSX)

S3 Table Stoichiometric matrix after modification to accommodate data. The original stoichiometric matrix in S1 was reduced to include only the reactions that transformed known, measurable metabolites to other known, measurable metabolites.
(XLSX)

S1 Code Precursor ratios used in synthesis reactions examining cartilage matrix synthesis. An explanation of the rationale for choosing precursors for collagen, aggrecan, lipids and albumin is given accompanied by the code used to compute the precursors.
(ZIP)

S4 Table Fluxes calculated from experimental data. Fluxes calculated using each of the biosynthesis reactions (e.g. type II collagen). Also included are the fluxes calculated without a biosynthesis reaction in the matrix.
(XLSX)

S5 Table A list of internal and external metabolites. This list details which of the measurable and constrained metabolites were classified as internal or external to the network, along with a short rationale for each.
(XLSX)

S6 Table Fluxes maximizing synthesis reaction flux. Fluxes calculated by solving a linear program for maximal for type II, VI collagen, aggrecan, albumin, and lipid biosynthesis using a stoichiometric matrix as constraints.
(XLSX)

S1 Fig Fluxes calculated with no biosynthesis reaction. The fluxes were calculated using a stoichiometric matrix that did not include a biosynthesis reaction. Fluxes calculated for time intervals (A) 0 to 15 min. and (B) 15 to 30 min. for compressed and uncompressed cells. Also included are fluxes calculated for (C) compressed cells for time interval 0 to 30 min. Reactions are represented by labeled boxes connected to substrates by incoming dark gray arrows and to products by outgoing dark gray arrows. Reaction fluxes are represented by arrows colored to match either positive (blue) or negative (yellow) flux. Larger arrows represent larger flux with linear scaling. Negative flux indicates a reversible reaction running in the direction opposite to the direction specified in the stoichiometric matrix. Electron transport chain and synthesis reactions not shown for simplicity.
(TIF)

S2 Fig LC-MS data for chondrocytes in response to 0–30 minutes of compression. Intensity values from LC-MS analysis of targeted metabolites for central energy metabolism. Note that these values were thresholded as described in the text prior to flux calculations.
(TIF)
