# Supplementary material for: Combining Targeted Metabolomic Data with a Model of Glucose Metabolism: Toward Progress in Chondrocyte Mechanotransduction
Source: PLoS One. 2017 Jan 5;12(1):e0168326. doi: 10.1371/journal.pone.0168326 (PMC5215894; doi:10.1371/journal.pone.0168326)
Supplement: S1 Code — An explanation of the rationale for choosing precursors for collagen, aggrecan, lipids and albumin is given accompanied by the code used to compute the precursors. (ZIP) [file pone.0168326.s004.zip › S4/S4_summary.pdf]

Determining Precursor Ratios

Precursor ratios of metabolites (i.e. the objective function used in solving for hypothetical fluxes) were determined from information in the NCBI database. NCBI provides amino acid composition and stoichiometric ratios of macromolecules in proteins that have a quaternary structure. A Python script was written that takes the protein name, stoichiometric ratio of the quaternary structure, and amino acid sequence of each protein copied directly from NCBI in the form of an ANSI encoded text file and produces the ratio of central metabolism precursors required to produce the necessary non-essential amino acids in the quaternary structure. The code first takes the amino acid composition from NCBI and removes any spaces and numbering. It then counts the occurrence of each amino acid and removes any essential proteins from the counts. It then compares the amino acids to a predefined dictionary in the code of the central metabolism precursors that correspond to the amino acids to produce a count of precursors. From this count, a ratio is calculated; this ratio was used as our objective function.

*This code is intended for use with Python 3. Using Python 2 might lead to compatibility issues.
