# Supplementary figures and images for: Combining Targeted Metabolomic Data with a Model of Glucose Metabolism: Toward Progress in Chondrocyte Mechanotransduction
Source: PLoS One. 2017 Jan 5;12(1):e0168326. doi: 10.1371/journal.pone.0168326 (PMC5215894; doi:10.1371/journal.pone.0168326)

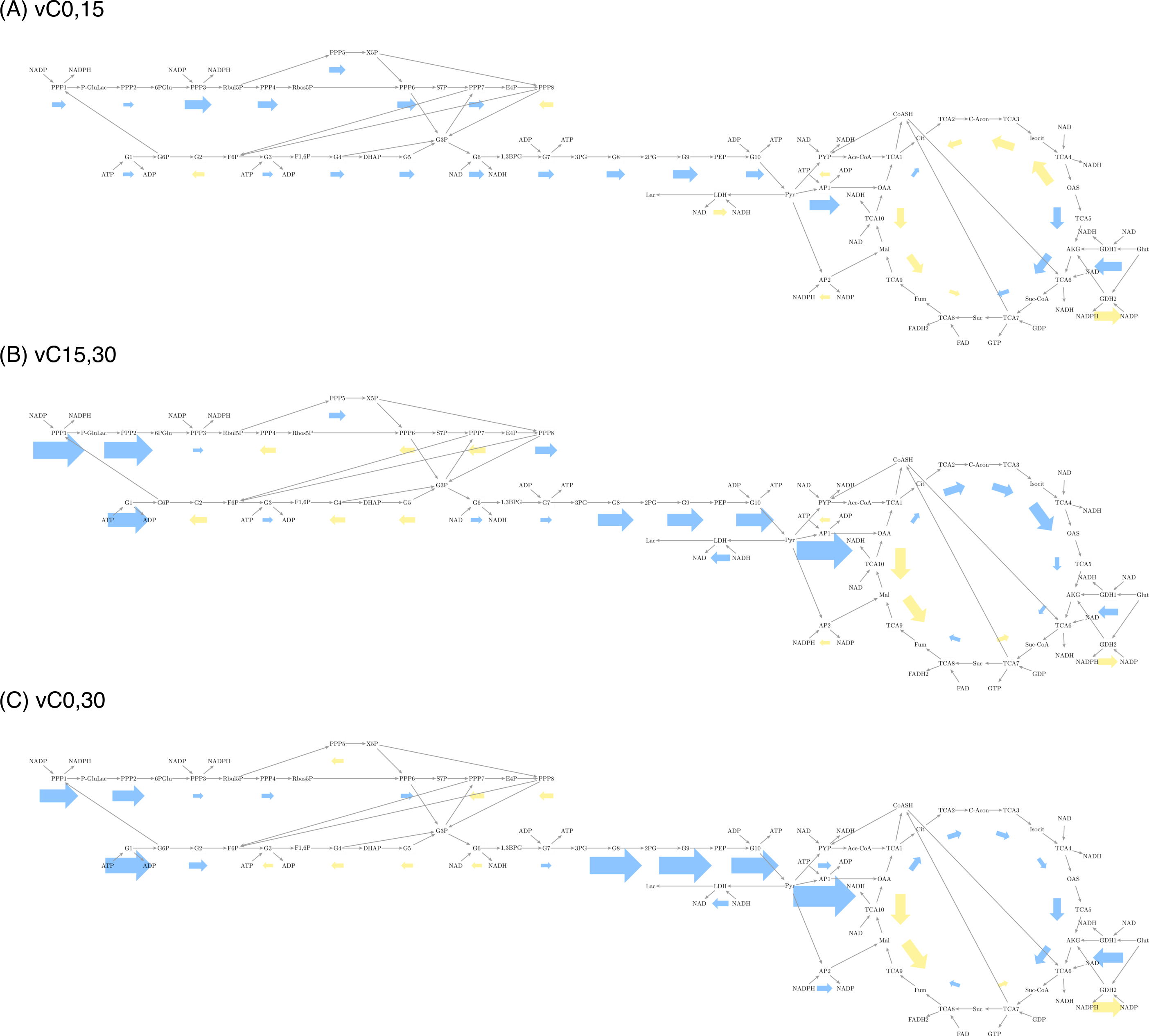

Supplement: S1 Fig — The fluxes were calculated using a stoichiometric matrix that did not include a biosynthesis reaction. Fluxes calculated for time intervals (A) 0 to 15 min. and (B) 15 to 30 min. for compressed and uncompressed cells. Also included are fluxes calculated for (C) compressed cells for time interval 0 to 30 min. Reactions are represented by labeled boxes connected to substrates by incoming dark gray arrows and to products by outgoing dark gray arrows. Reaction fluxes are represented by arrows colored to match either positive (blue) or negative (yellow) flux. Larger arrows represent larger flux with linear scaling. Negative flux indicates a reversible reaction running in the direction opposite to the direction specified in the stoichiometric matrix. Electron transport chain and synthesis reactions not shown for simplicity. (TIF) [file pone.0168326.s008.tif]

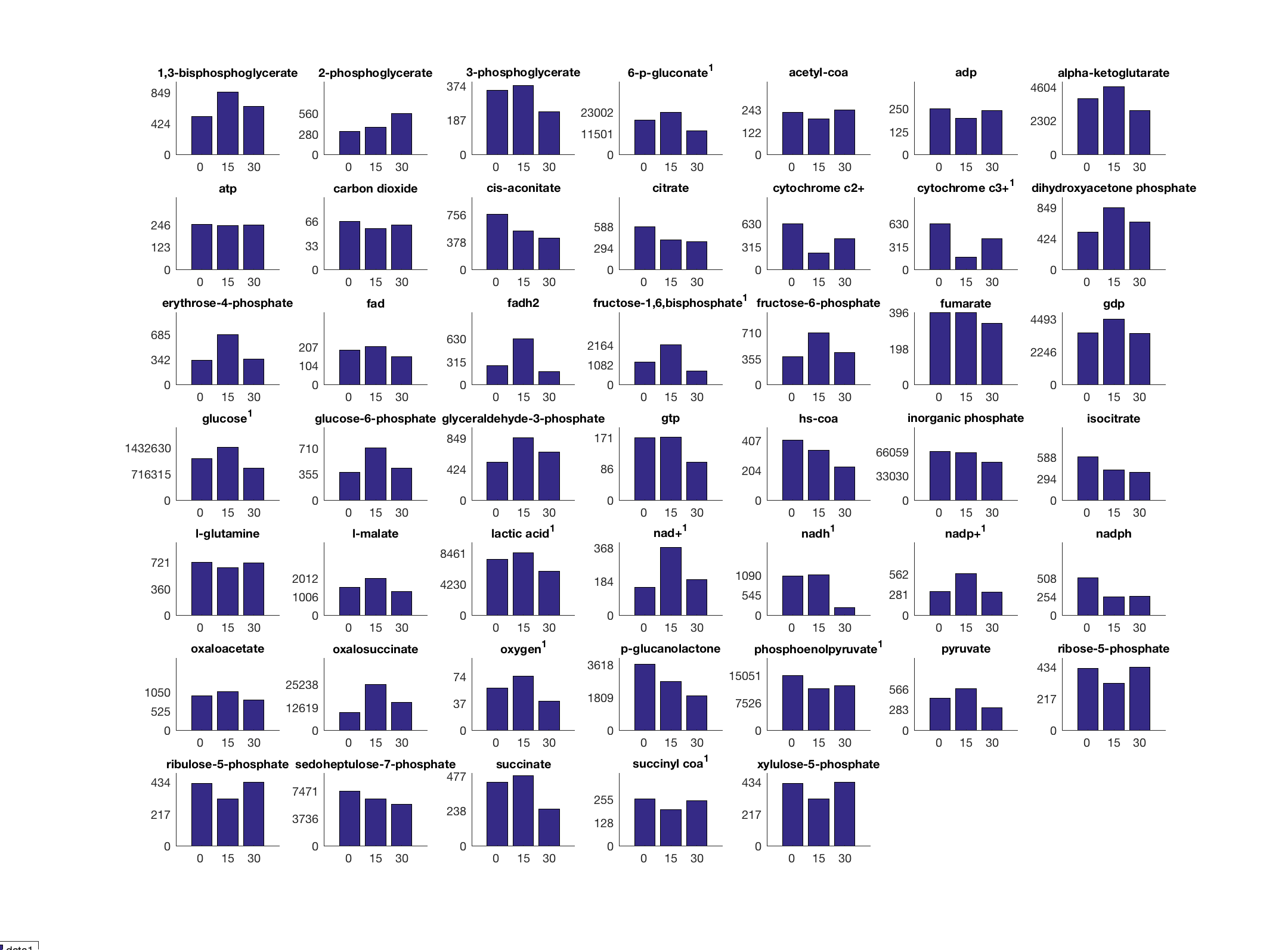

Supplement: S2 Fig — Intensity values from LC-MS analysis of targeted metabolites for central energy metabolism. Note that these values were thresholded as described in the text prior to flux calculations. (TIF) [file pone.0168326.s009.tif]
